# Sinus Augmentation by Platelet-Rich Fibrin Alone: A Report of Two Cases with Histological Examinations

**DOI:** 10.1155/2016/2654645

**Published:** 2016-10-03

**Authors:** Naofumi Aoki, Takeo Kanayama, Michinori Maeda, Koichiro Horii, Hironori Miyamoto, Keinoshin Wada, Yasutaka Ojima, Tsukasa Tsuchimochi, Yasuyuki Shibuya

**Affiliations:** ^1^Department of Oral Maxillofacial Surgery, Graduate School of Medical Sciences, Nagoya City University, Nagoya 467-8601, Japan; ^2^Hakata Dental Clinic, Fukuoka 812-0011, Japan

## Abstract

In sinus floor augmentation of an atrophic posterior maxilla, platelet-rich fibrin (PRF) has been used as a graft material. We herein report two cases with histological evaluations of PRF after the surgery. The first case was a 28-year-old female with an atrophic right posterior maxilla who was treated with sinus floor augmentation and simultaneous implant placement using PRF as the sole graft material in our hospital. Twenty-four months after surgery, the implant was unfortunately removed because of occlusal overloading by parafunctional habits. During implant replacement, a tissue sample was obtained from the site of augmentation with PRF and was evaluated histologically. The second case was a 58-year-old man with severe alveolar atrophy of the right maxilla who underwent lateral sinus augmentation using only PRF in a two-stage procedure in our hospital. Samples were obtained at the second-stage surgery and histological examinations were performed. As a result, new bone formation was confirmed histologically in both cases. Our findings show that the use of PRF as a graft material during sinus floor augmentation induces natural bone regeneration.

## 1. Introduction

In the posterior maxillary area, insufficient vertical height of the alveolar bone due to the presence of the maxillary sinus, postextraction bone resorption, and poor quality and quantity of alveolar bone may limit implant placement [1]. In such cases, several sinus augmentation procedures for implant placement have been introduced since the 1980s [2, 3]. The techniques create space between the maxillary alveolar process and the elevated Schneiderian membrane, which is filled with various graft materials to maintain adequate space for new bone formation. Many graft materials have been applied to these techniques such as autogenous bone, allograft, xenograft, alloplastic bone, or combinations thereof [4, 5]. Although autogenous bone is considered to be the gold standard, it creates another wound at the donor site. Thus, autogenous bone is not widely used in clinical practical. The other graft materials also have limitations, including risk of infection, insufficient bone regeneration, and increased overall cost. Accordingly, no graft material appears to be superior to the others.

Platelet-rich fibrin (PRF) was first reported in 2001 by Choukroun et al. as a second-generation platelet concentrate [6]. Several studies have recently reported the application of PRF in dental implant surgery. Notably, PRF mixed bone substitute or PRF has so far solely been used as a graft material for sinus augmentation using both the lateral and crestal approaches [7–9]. The application of PRF for sinus augmentation is a relatively easy surgical procedure and the clinical and radiological findings have been shown to have a good effect regarding new bone formation. However, the histological findings of patients treated by this method have not frequently been investigated because it is difficult to harvest such augmented tissue for histological evaluations. We herein experienced two patients who exhibited sinus augmentation with PRF as the sole graft material and discussed the histological findings after sinus augmentation.

## 2. Case Presentation

We herein report the findings for two patients who underwent sinus floor augmentation using PRF as the sole graft material at the Department of Oral and Maxillofacial Surgery, Nagoya City University Hospital, Japan. The clinical procedures were performed in accordance with the Helsinki Declaration (revised in 2008) and approved by the Ethics Committee of Nagoya City University Hospital, Japan (number 46-10-0004). Both patients signed an informed consent form before treatment.

PRF is easy to prepare and cheap and has wound-healing properties for sinus membrane and bone. The focus when forming new bone in the sinus is on maintaining the Schneiderian membrane rather than promoting new bone growth. PRF may be absorbed more rapidly after surgery compared to its ability to form new bone. However, the Schneiderian membrane after sinus floor elevation with simultaneous implant placement can be maintained in a tent-like state, as the implant apexes function as “tent struts.” We decided to use PRF as a graft material because PRF has wound-healing properties and physically protects the sinus membrane during simultaneous implant placement. In addition, should PRF alone penetrate into the sinus, there is a lower risk of sinus infection than if PRF mixed with graft materials such as allografts and xenografts penetrated the sinus. Therefore, we decided to use only PRF as a graft material.

### 2.1. Case 1

**Figure 1:**
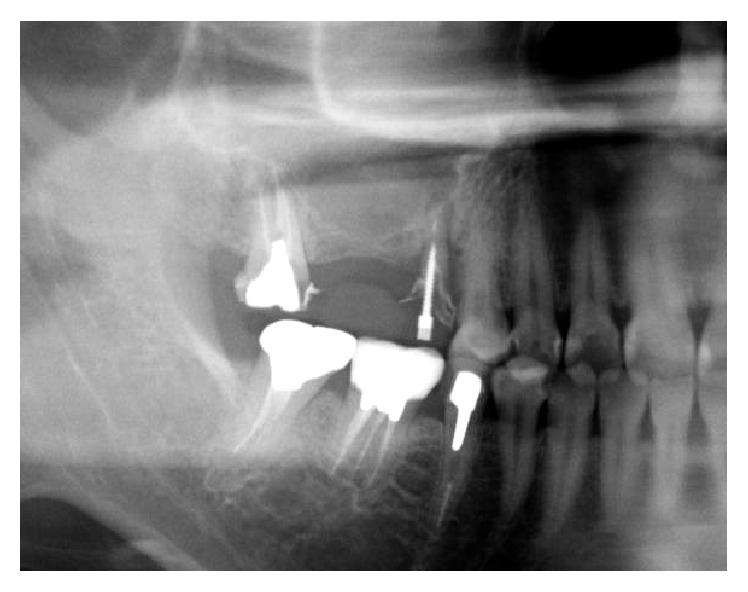
A panoramic radiograph reveals the residual bone height to be low at the first molar site.

In September 2010, a 28-year-old woman was referred to our hospital complaining of loss of retention of a fixed bridge in the right maxillary molar region. The second premolar had a poor right condition and the first molar was missing. Her general health was good and she was a nonsmoker. Following second premolar extraction, she required dental implant therapy to replace her missing teeth. An initial radiographic examination showed that the alveolar bone height of this area was insufficient for implant placement (Figure 1). Preoperative computer tomography (CT) revealed that the residual bone height between the alveolar crest and the maxillary sinus floor was 2.7 mm in the second premolar region and 2.2 mm in the first molar region. Therefore, sinus floor elevation was planned. The operation was performed using the crestal approach using PRF as the graft material. Before grafting, PRF clots were compressed into a thin membrane according to the protocol by Choukroun et al. [6], and multiple PRF membranes were inserted into the sinus floor elevated site. Two implants (KYOCERA, Osaka, Japan; POIEX: diameter, 42 mm, and length, 10 mm, and diameter, 47 mm, and length, 8 mm) were simultaneously placed using the osteotome technique within the sinus augmentation area. At 6 months postoperatively, second-stage surgery was performed and screw-retained crown rehabilitation was conducted.

**Figure 2:**
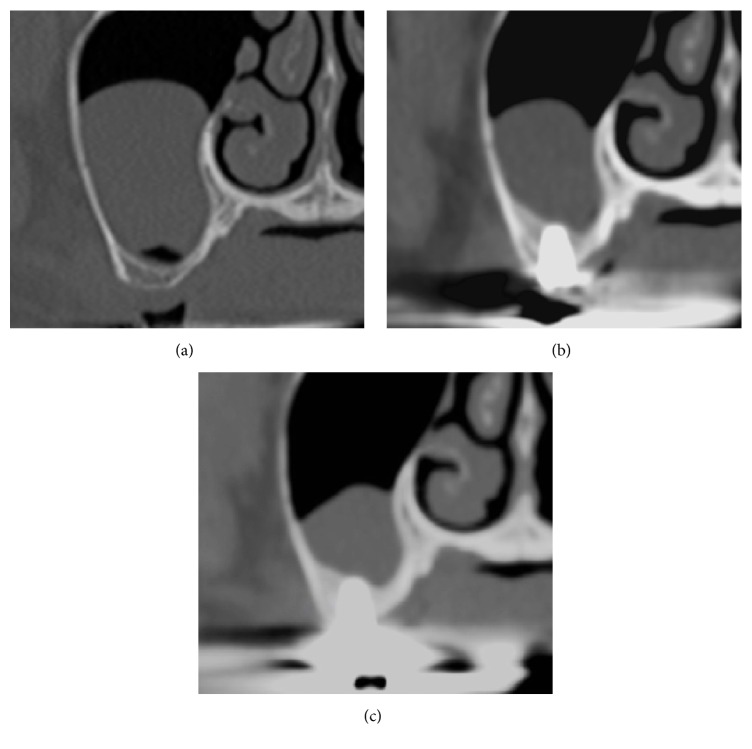
CT scans were performed and showed the implant (site 16). (a) A preoperative image shows the residual bone height is approximately 2 mm. (b) Six months after surgery, the original sinus floor was confirmed. Furthermore, gradually increasing radiopacity was observed around the implant up to the apex, which is the elevated site using PRF only. (c) One year after surgery, the original sinus floor is unclear and radiopacity around the implant increased.

**Figure 3:**
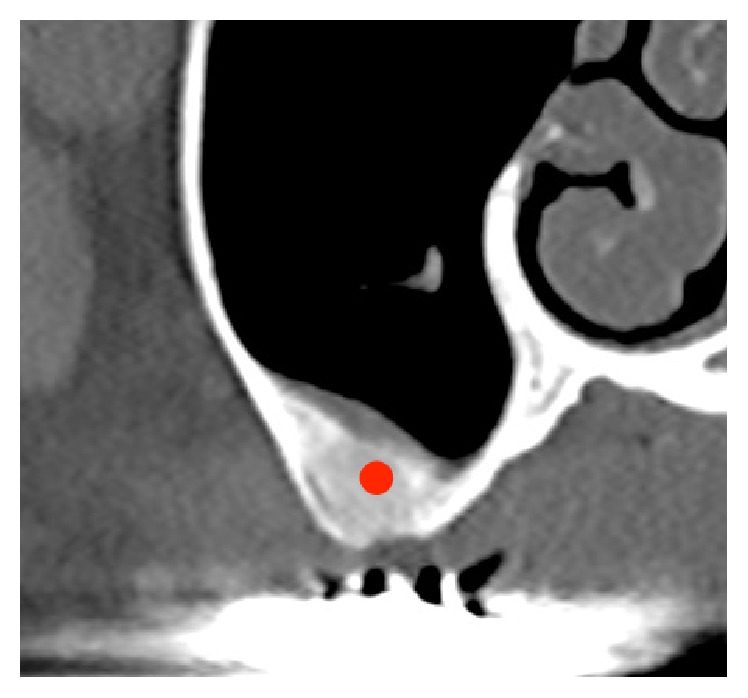
A biopsy was harvested from the red dot, which indicates the site of newly formed tissue (Case 1).

At 12 months postoperatively, CT of the first molar region showed increasing radiopacity within the sinus augmentation area around the implant (Figure 2). However, the implant in the first molar region showed mobility and was unfortunately removed at 24 months after surgery. The reason for this loss was considered to be occlusal overloading by parafunctional habits. She again required implant treatment; therefore the implant was reinserted using the CAS-KIT (Osstem Implant Co., Busan, Korea) in the slightly mesial area of the removed implant. According to the manufacturer's guideline, the CAS-drill must be used with a stopper. Using the stopper, the drill can collect a bone chip precisely from the depth of the settling zone within the drill hole. Accordingly, a bone chip within the sinus augmented area that was above the original sinus floor line was harvested using the CAS-drill with a 9 mm stopper (Figure 3). After drilling with a final stopper, which penetrated just until the sinus floor, the sinus membrane was lifted using hydraulic pressure and an implant (Osstem Implant Co., Busan, Korea; TSIII: diameter 5.0 mm and length 8.5 mm) was placed. Crown rehabilitation was performed 8 months after the reinsertion surgery. Samples were stained with H-E and analyzed with a light microscope (Olympus Co., Tokyo, Japan: BX51).

### 2.2. Case 2

**Figure 4:**
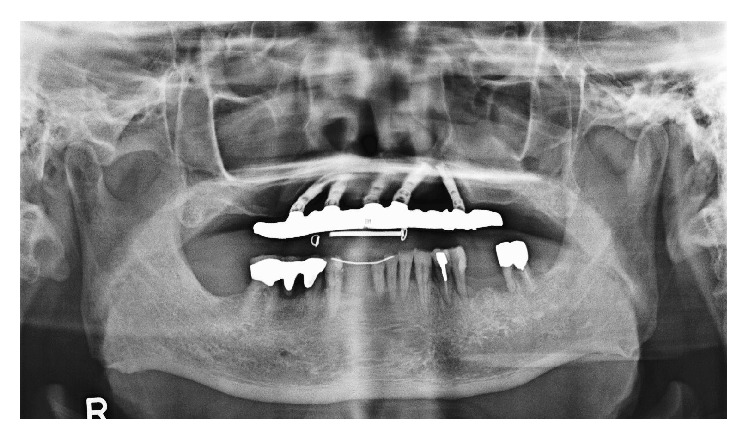
The initial panoramic radiograph revealed severe bone loss around R3 and L2 implants.

**Figure 5:**
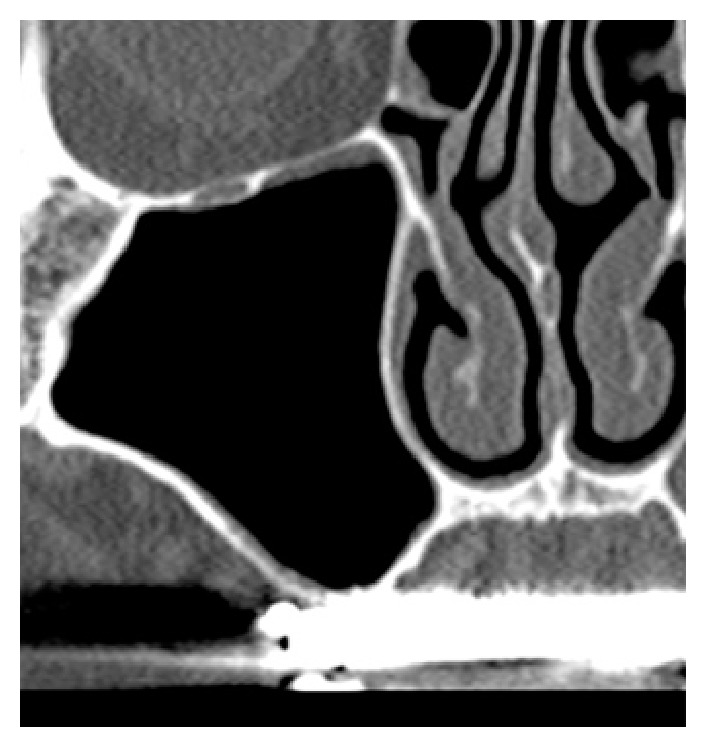
The preoperative CT scan showed insufficient residual height in the right posterior area (Case 2).

**Figure 6:**
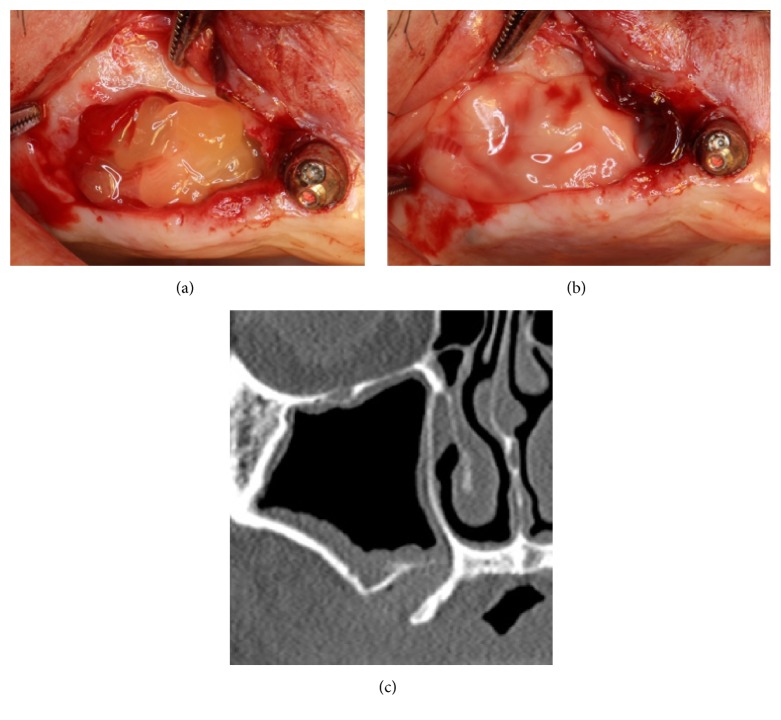
Sinus augmentation using the lateral window technique. (a) PRF clots were filled inside the sinus cavity and (b) the PRF membrane was used to cover the osteotomy window. (c) A CT image taken immediately after sinus augmentation using PRF only.

**Figure 7:**
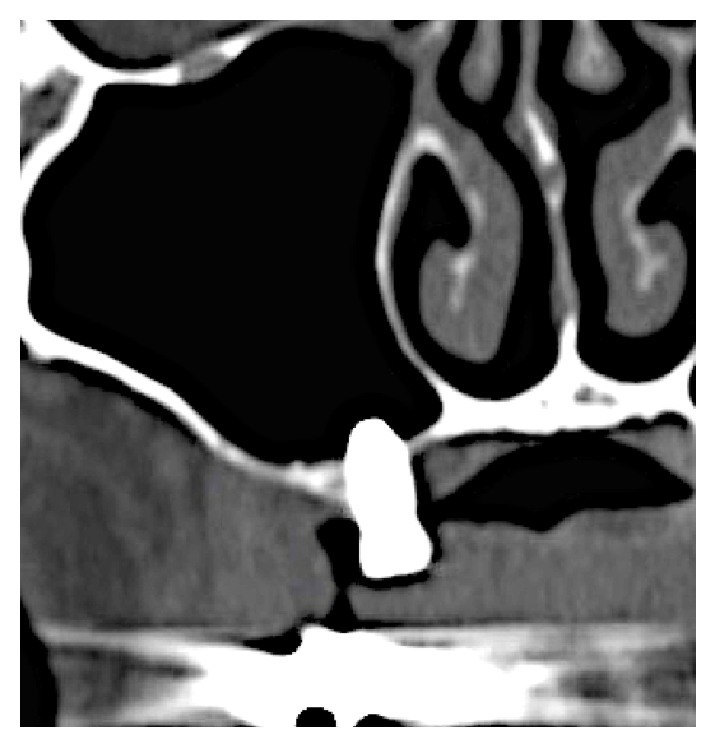
CT scan 8 months after sinus augmentation in the first stage showed that radiopacity around the implant increased.

In January 2014, a 58-year-old man was referred to our hospital from a general practitioner for implant removal because of peri-implantitis. Five implants and a fixed prosthesis had been placed in the edentulous maxilla 7 years previously. He showed no systemic pathology and he was a smoker. An initial radiographic image revealed severe bone loss around the R3 and L2 implants (Figure 4). Therefore, we decided to remove the implants and place additional implants into the molar area. As the bone height under the sinus in the right posterior area was less than 2 mm (Figure 5), the lateral window technique was chosen. First, the R3 implant was removed, the simulation sinus was elevated using the lateral window technique, 2 PRF clots were inserted inside the sinus cavity, and a PRF membrane was used to cover the osteotomy window (Figure 6). Three months after sinus augmentation, a tissue biopsy using the CAS-KIT for implant placement was harvested from the grafted site using PRF and implants (Osstem Implant Co., Busan, Korea; TSIII: diameter, 4.5 mm, and length, 8.5 mm) were placed using the crestal approach. Five months after surgery, the implants were osteointegrated and restoration occurred (Figure 7). The biopsied samples were stained with toluidine blue and analyzed with a light microscope (Olympus Co., Tokyo, Japan: BX51).

## 3. Histological Examination

**Figure 8:**
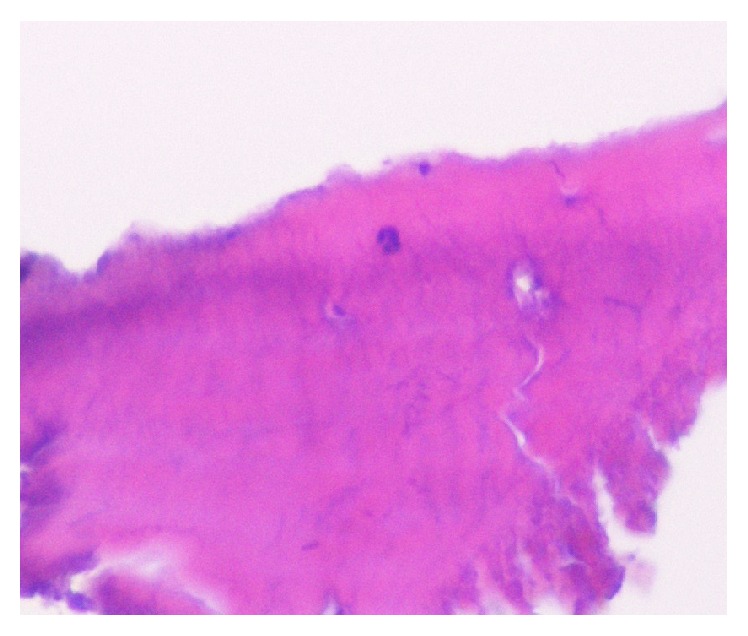
Histological examination in higher magnification of a sample harvested 24 months after surgery using PRF only shows extremely mature bone tissue (H-E staining).

**Figure 9:**
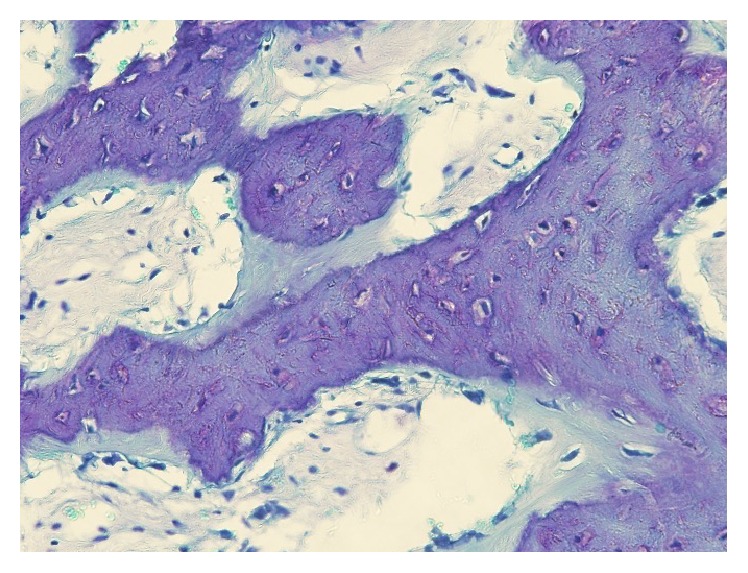
Under high magnification, newly formed lamellar bone surrounding the woven bone was confirmed (toluidine blue staining).

The tissue samples from Case 1 showed lamellar structures including osteocytes. Extremely mature bone tissue was observed without inflammatory cells, osteoblasts, or osteoclasts (Figure 8). The tissue samples from Case 2 showed woven bone around mature lamellar bone (Figure 9).

## 4. Discussion

Recently, the application of autologous platelet concentrates to enhance bone regeneration has increased in maxillofacial surgery [10]. The use of platelet-rich plasma (PRP) was first reported in oral surgery procedures [11]. However, there are potential risks with this material, as PRP contains synthetic or anticoagulant materials. On the other hand, PRF developed by Choukroun et al. does not require heterogeneous agents. PRF is preferred to PRP because PRF is safer and cheaper to prepare, and the production time is shorter [12]. Furthermore, PRF has advantages over PRP in biological activation. PRF contains more growth factors than PRP and releases these growth factors gradually. The growth factors are expressed more strongly and exert a more durable effect on proliferation and differentiation than PRP [13]. However, according to Arora et al., it is difficult to assess the practical benefits of PRP in maxillary sinus augmentation procedures [14].

Previous studies have reported sinus augmentation using PRF in combination with conventional bone substitute. Choukroun et al. reported sinus floor augmentation using PRF in combination with a freeze-dried bone allograft [15]. In their report, histological evaluations revealed that sufficient new bone formation was observed. Moreover, the mixed graft material could reduce the healing time. In other studies, PRP was added to the bone graft used for sinus augmentation and evaluated histologically. Raghoebar et al. reported no beneficial effect of PRP on wound healing or bone remodeling [16]. Tanaka et al. demonstrated that increased new bone formation could be found in the histological evaluation of sinus augmentation with deproteinized bovine bone mixed with PRF [17]. However, these granular materials are associated with increased costs and risk of infection.

Accordingly, sinus floor elevation using PRF as the sole graft material has been recently introduced. Simonpieri et al. followed up a case of dental implant treatment combined with simultaneous sinus lift with PRF as the sole filling material and demonstrated that stabile new bone formation occurred around the dental implants [18]. Diss et al. also showed that, in cases with a mean residual alveolar bone height of 6 mm, the implants could function in new bone after 1 year from sinus augmentation with PRF used as the sole filling material [8]. Furthermore, more severe cases were applied in our previous report, in which PRF was used as the sole material in cases of atrophic maxilla with residual bone height of <5 mm and endosinus bone gain was promoted using the crestal approach in sinus floor elevation [19]. Additionally, PRF was placed around implants and assessed at about 6 months after surgery using radiography and CT, and the results showed sufficient newly formed bone [20]. However, thus far, no study has yet conducted a histological examination of PRF as the only graft material after sinus augmentation.

There are a few reports about histological evaluations of PRF after surgery. Mazor et al. examined histology tissue 6 months after a sinus lift and demonstrated that the tissue was harvested from a lateral window region using a trephine drill [21]. Biopsies at 6 months after the sinus lift procedure showed a high volume of natural bone with osteoblasts and osteocytes identified easily. However, Case 1 showed bone tissue without osteoblasts. The samples were harvested after a relatively long time had passed since surgery and showed extremely mature bone tissue. In that report, it was not clear whether the regenerating bone closely originated from the implant site or another site.

In this report, a one-stage protocol in which implants were simultaneously placed with sinus augmentation was employed in Case 1, and a tissue sample was obtained more than two years after the protocol. The sample was harvested from a site that was clearly localized beyond the original sinus floor line; therefore this area was considered to be newly formed bone tissue. On the other hand, a two-stage protocol (sinus augmentation in the first stage and delayed implant placement in the second stage) was employed in Case 2 because the residual bone height in Case 2 was less than 2 mm. Additionally, in the first stage of Case 2, PRF was applied as the sole graft material. To the best of our knowledge, this is the first report using PRF alone in a two-stage protocol of sinus augmentation. Several graft materials have been introduced for the protocol to promote bone regeneration [4, 22]. In particular, PRF has the ability to release growth factors, such as platelet-derived growth factors and transforming growth factor-beta, and is considered to be a healing biomaterial with great potential for bone regeneration.

Some reports have argued that graft materials are not necessary during sinus floor elevation [23–25]. Nedir et al. suggested that graft materials were not necessary at residual bone heights >4 mm [26]. However, a long-term evaluation of sinus floor augmentation without graft materials has not yet been conducted. Another crucial factor in the success of sinus augmentation without graft materials is maintaining an elevated Schneiderian membrane. In the one-stage protocol, implant apexes are responsible for maintaining the membrane at an adequate height from the original sinus floor line, such as a tent peg [27, 28]. Conversely, in the two-stage protocol, graft materials are responsible for maintaining the Schneiderian membrane position. PRF is absorbed gradually; therefore, PRF as the sole filling material without simultaneous implant placement may not be adequate for maintaining the membrane [20]. However, our findings in the present case report dispute this notion. Indeed, Case 2 demonstrated that PRF clots alone have the capacity for creating space between the original sinus floor and the elevated Schneiderian membrane and the space could be confirming histologically new bone formation. The volume of PRF may be important for maintaining the membrane. Therefore, PRF clots may be considered more favorable than a PRF membrane. Our study describing this case with the two-stage protocol has limitations like short follow-up period. Further studies are therefore needed to obtain stronger evidence to confirm our findings.

## 5. Conclusion

We reported two cases in which PRF used as the sole graft material for sinus augmentation was able to be displaced by new bone. Based on these findings, at 24 months after surgery, the use of PRF alone should be sufficient to maintain natural new bone around protrusion implants into the sinus. The two-stage protocol with PRF only is a safer and simpler technique than the protocol with graft materials mixed with PRF; however, the ability of PRF to form and maintain new bone under the two-stage protocol remains unclear. Although PRF as the sole graft material is useful because it can be easily obtained, is cost-effective, and has the potential to promote natural bone regeneration, further studies with more cases will be required.
